# Honey Bee Antiviral Immune Barriers as Affected by Multiple Stress Factors: A Novel Paradigm to Interpret Colony Health Decline and Collapse

**DOI:** 10.3390/v10040159

**Published:** 2018-03-30

**Authors:** Francesco Nazzi, Francesco Pennacchio

**Affiliations:** 1 Dipartimento di Scienze AgroAlimentari, Ambientali e Animali, Università degli Studi di Udine, 33100 Udine, Italy; 2 Dipartimento di Agraria—Laboratorio di Entomologia “E. Tremblay”, Università degli Studi di Napoli “Federico II”, 80055 Portici (Napoli), Italy; f.pennacchio@unina.it

**Keywords:** deformed wing virus, microbiota, multiple interactions, mutualistic symbiosis, NF-κB, nutrition, *Varroa destructor*

## Abstract

Any attempt to outline a logical framework in which to interpret the honey bee health decline and its contribution to elevated colony losses should recognize the importance of the multifactorial nature of the responsible syndrome and provide a functional model as a basis for defining and testing working hypotheses. We propose that covert infections by deformed wing virus (DWV) represent a sword of Damocles permanently threatening the survival of honey bee colonies and suggest that any factor affecting the honey bee’s antiviral defenses can turn this pathogen into a killer. Here we discuss the available experimental evidence in the framework of a model based on honey bee immune competence as affected by multiple stress factors that is proposed as a conceptual tool for analyzing bee mortality and its underlying mechanisms.

## 1. The ‘Damocles Sword’ of Honey Bees: Viral Infections and Colony Losses

Beekeeping, in its long-lasting history, has gone through several crises, which caused either great concern or notable damage [1]. In some case, they remained for long time as mysterious accidents, which were unraveled only after the puzzling problems behind these events were properly addressed [2].

The latest and not yet resolved crisis has had a relevant negative impact on the world of managed bees in the last decade and can certainly be regarded as one of the most problematic. It is associated with a new syndrome reported both in North America and many other countries in the Northern hemisphere [3,4], generating a dramatic scenario of problematic diagnosis [5]. This stimulated an unprecedented research effort aiming to unravel the possible causes of this phenomenon, that has generated a remarkable body of knowledge, roughly summarized as follows: (i) a 20–30% colony losses are regularly registered over the autumn–winter period in most countries of the Northern hemisphere [3,4]; (ii) no single cause can be claimed as responsible for this phenomenon but a number of stress factors are involved [6,7], among which bee viruses and, in particular, the deformed wing virus (DWV), seem to play a major role [8,9,10].

The dramatic impact of DWV infections, often leading to colony collapse, is not the consequence of a biological invasion by an aggressive novel pathogen, just starting a co-evolutionary arm-race with local bees. Indeed, DWV has long been endemically present as a harmless pathogen, able to develop asymptomatic covert infections [11]. The limited acute effects of DWV infections have favored its capillary spread to virtually all honey bee colonies, dramatically fostered by the vectoring activity of the parasitic mite *Varroa destructor* [12], which, instead, can be regarded as a novel parasite for *Apis mellifera*, having invaded the Western World in the second half of the last century, after a crucial host switch from *Apis cerana* [13]. The mite is undoubtedly one of the major agents which, in synergy with other stressors promotes uncontrolled viral replication and the transition of common covert infections to devastating overt infections [9].

It is therefore clear that DWV covert infections are not due to a low pathogenicity of the virus but rather to the fact that antiviral barriers are able to contain viral infection in honey bees when immuno-competence is not impaired by external stress agents [6].

**Figure 1:**
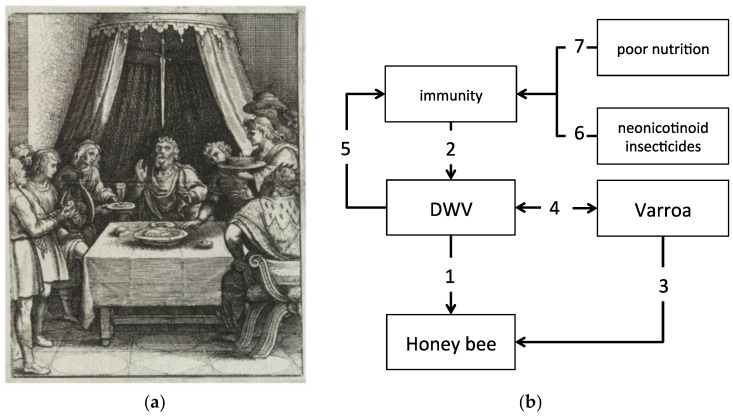
The expression ‘sword of Damocles’ denotes the sense of foreboding engendered by a precarious situation, whereby the onset of tragedy is restrained only by a delicate trigger. (**a**) A pictorial representation of the legend that generated the colloquial expression, by Wenceslaus Hollar (1607–1677), where the sword hanging above Damocles neck is depicted; (**b**) Honey bees are permanently threatened by covert DWV infections (1) that are normally kept under control by the bee’s immune system (2); the *Varroa* mite, which also causes direct damage to bees (3), can trigger viral replication and take advantage (4) of the immune suppression exerted by the virus above a certain threshold (5). Other stressors, such as neonicotinoid insecticides (6) or poor nutrition (7) can interfere with the system by impacting either directly or indirectly honey bee immunity.

A metaphor for this situation is described in a classic legend that generated the common colloquial expression ‘sword of Damocles’ and refers to the story of a courtier of Dionysius II of Syracuse, a fourth-century BC tyrant of Syracuse in Sicily [14]. According to this legend, Damocles was offered to switch place with his king Dionysius for one day, in order to experience the fortune of king’s life. However, soon after eagerly accepting this offer, Damocles realized that a huge sword was hanging above the throne, held by a single hair of a horse’s tail (Figure 1a). Therefore, this expression is often used to denote the sense of foreboding engendered by a precarious situation, in which the onset of tragedy is restrained only by a delicate trigger or chance.

In view of the current knowledge about the dangerous interactions between honey bees and DWV, we can consider covert DWV infections as a kind of ‘sword of Damocles’, permanently threatening the bee colony survival, as any stress factor, or combination thereof, may weaken the resistance of that thin hair, the equivalent of bee’s antiviral defense barriers, and can have dramatic consequences (Figure 1b). For this reason, understanding how honey bees deal with viruses, and in particular DWV, and how other stressors can influence this interaction is crucial for formulating a functional framework to study bee health comprehensively.

## 2. The Immune Model

The multifactorial nature of honey bee health decline and its contribution to elevated colony losses became evident when a wealth of studies focusing on specific stressors failed to demonstrate a direct causal link universally accounting for bee health decline and colony loss induction. This generated a general consensus on the hypothesis that no single factor can be invoked as the very cause of colony decline and eventual loss, which are in most cases associated with different combinations of stressors [7]. However, unraveling the complex dynamics underlying the synergistic interactions among different stress agents is not trivial and is still far from being completely achieved. Studies have been particularly intense on the association between the *Varroa* mite and the vectored viral pathogens, which were soon recognized as key players in the intricate network of interactions underlying honey bee colony mortality.

In 2012, in the framework of a field study aiming at exploring the role of the parasitic mite *V. destructor* in the collapse of honey bee colonies, we exposed bee colonies to an increasing parasitic pressure in order to cause a controlled collapse and explore the underpinning mechanistic factors [9]. A clear negative correlation between mite infestation and colony strength was evident, but the causal link accounting for this observation appeared to be the increased replication of DWV, triggered by the mite, which had a severe impact on honey bee survival and eventual colony demise. The molecular analyses clearly indicated that the immune system of honey bees is of central importance in the modulation of this tripartite interaction. Briefly, a combination of field and lab experiments revealed that the delicate balance keeping the covert viral infections under control was disrupted by an immuno-suppressive syndrome, characterized by a negative transcriptional regulation of several genes, among which the strongest effect was observed on *dorsal 1A* [9], a transcription factor in the family NF-κB [15,16], controlling a number of immune barriers, including those against viruses under the Toll pathway [17,18,19,20,21,22]; in fact, RNAi mediated silencing of this gene was clearly associated with increased viral replication [9]. The importance of these poorly characterized antiviral barriers under NF-κB control has been further corroborated by the fact that the virulence strategy adopted by DWV largely relies on negative regulation of this transcription factor [23]. Later, a significant downregulation of *dorsal* was observed also in other studies on mite infested/virus infected honey bees [24,25] and the role in antiviral defense was confirmed in *Drosophila* [26].

This negative regulation of NF-κB by DWV reduces clotting and melanization in infected honey bees and promotes a fitness gain for the mite, which reproduces at higher rates on virus infected bees as a likely consequence of a more efficient food uptake and use [23]. The resulting higher feeding pressure by the mite on infected bees promotes a more intense viral replication, by activating a number of competing immune reactions and stress responses [9]. This type of mutual benefit between the mite and DWV can be regarded as a symbiotic association, which results in a positive feedback loop with devastating consequences [23].

The honey bee immuno-competence is then heavily influenced by this mite-DWV symbiotic interaction, and can be seen as the thin hair suspending the Damocles’ sword of viral infections threatening the bee colony: any factor stressing this hair can let the sword fall.

## 3. Testing the Model: The Case of Clothianidin

The conceptual framework outlined above allows to predict that any additional stress agent with a negative impact on antiviral immunity has the potential to boost an uncontrolled viral replication. This hypothesis can be experimentally tested by modulating the immuno-competence of honey bees.

Among the many factors that can potentially have this effect, we focused our attention on neonicotinoid insecticides, as they were reported to have a negative effect on the expression of a NF-κB transcription factor in the mussel *Mytilus galloprovincialis* [27]. This background information paved the way for the first in vivo testing of the Damocles sword model of the interactions. By using a combination of molecular tools and *Drosophila* as a model system, it was possible to demonstrate that the neonicotinoid clothianidin negatively modulates NF-κB activation and promotes DWV proliferation in infected honey bees exposed to sub-lethal doses of this insecticide [28]. These results do not support the claim that neonicotinoids are responsible for honey bee colony decline and collapse, but provide unequivocal evidence that they can negatively affect bee immuno-competence, contributing to the chronic decay of bee colonies exposed to contaminated vegetation. Then, depending on the number of immuno-active stressors to which honey bees are exposed, colony strength and the starting viral infection level, the effect of neonicotinoids on viral proliferation can be different from case to case. However, this does not reflect the inconsistency of the described immuno-modulative effect of neonicotinoids, but rather the variability of the combination of stress agents that can change over space and time.

## 4. Model Testing at Higher Complexity Levels

The central role of the immune system in the modulation of mite-virus interaction allows us to predict that any external factor affecting immune barriers has the potential to further exacerbate the negative effects of the *Varroa*-DWV mutualistic symbiosis.

In particular, the negative effect of clothianidin on NF-κB signaling [28], which also occurs in vertebrates [29], is similar, in functional terms, to that exerted by DWV [9,23]. Therefore, the immuno-suppressive role of clothianidin, disrupting clotting and melanization, may, in principle, promote an increase of *Varroa* fitness; this would lend support to previous field work, suggesting a subtle effect of neonicotinoid exposure on mite proliferation and associated prevalence of viral pathogens [30]. Indeed, these two aspects, for the reasons discussed above, cannot be completely separated, since they are part of a unique response that has additional layers of complexity not yet fully understood.

However, the negative impact of neonicotinoids on insect immunity is not only due to their direct effects on the network of molecular signaling governing stress responses, but is also mediated by subtle mechanisms acting at the meta-organism level. Indeed, it has been recently demonstrated that imidacloprid, by interfering with immuno-modulatory mechanisms regulating the gut microbiota, reduces the capacity of *D. melanogaster* larvae to face pathogen infections [31]. Moreover, this negative effect can be compensated by the administration of bacterial species alleviating the dysbiosis induced by neonicotinoids [31]. These results shed light on how important the microbiota is in the modulation of immune response in insects, even in the case of antiviral barriers, which are dependent in part by gut microorganisms [32]. The physiological importance of the honey bee microbiota and its central role in the modulation of the immune barriers is already fully appreciated [33,34,35]; it will have to be carefully taken into consideration for a correct interpretation of the impact of insecticides on immuno-competence of bees and on their capacity to cope with interacting stressors acting upon the stability of the mite-virus association.

## 5. The Implications of Nutrition and Nutritional Immunology

The cross-modulation between immunity and nutrition is a very active research area. There is increasing evidence of the multifaceted aspects of nutritional immunology, which has remarkable physiological, ecological, and evolutionary implications [36]. Accumulating experimental data on honey bees strongly support the view that nutrition is implicated in the modulation of the intricate network of metabolic pathways, which cross-modulate bee immunity [37]. Indeed, a number of genes belonging to metabolic pathways show an altered expression pattern in response to mite infestation coupled with DWV infection [25]. Moreover, the supply of pollen to such bees extends longevity and reduces viral infection [38] through mechanisms which are currently being investigated.

These complex metabolism-mediated mechanisms of immune control can be even more complicated when parasites and pathogens are added into this system [37]. Indeed, nutrition appears to play a role in the regulation of the stability of bee–mite–DWV triangle. We recently determined that the hemolymph of DWV-infected bees contains a greater amount of proteins, as compared to unaffected bees [39]. This result indicates that DWV infections may modulate bee metabolism further reinforcing the viral support to mite’s fitness, far beyond the simple increase of food use efficiency associated with immuno-suppression [23]. This is not an unusual evolutionary pattern in insect parasitology. Indeed, the host regulation by parasitic insects is performed with the help of viral symbionts, which are able to modulate host immunity, metabolism and reproduction, in order to facilitate its exploitation by their developing progeny [40]. The wealth of physiological and nutritional interactions between parasitic insects and their hosts indicate the occurrence of intricate patterns of co-evolution in these tripartite associations with microbial symbionts, which are still poorly known in the case of bees and their cohort of parasites and pathogens. Unraveling the functional bases governing these intricate associations is certainly worth of further research efforts and the molecular tools currently available can open up exciting perspectives for mechanistic studies [41].

## 6. Conclusions and Future Perspectives

The comprehension of the intimate molecular aspects of DWV-mite interaction, which is the core of the Damocles sword paradigm, is far from being complete. The analysis of the functional elements underlying the symbiotic association between *Varroa* and DWV sheds light on additional aspects, which have been so far largely overlooked. An even more complex framework is generated when these complex interactions are analyzed by taking into account the important role of the honey bee microbiota.

In insects, as in all living organisms, a number of physiological and pathological pathways can be fully understood only at a metaorganism level, since the associated microbiota plays a key role in their regulation. Therefore, as already suggested [34], all future studies on honey bees will have to take into account this important aspect, which adds an intriguing layer of complexity to the mechanisms governing the life of this social insect. Upgrading the immune model we proposed, based on the complex synergistic interactions among different stress agents, to the metaorganism level makes things even more complicated. A realistic analysis of the emerging multifaceted scenario can be properly performed, only adopting a systems biology approach, towards a more comprehensive and efficient modeling as a basis for rationale management and protection of honey bee colonies.
